# Aesthetic Treatment Outcomes of Capillary Hemangioma, Venous Lake, and Venous Malformation of the Lip Using Different Surgical Procedures and Laser Wavelengths (Nd:YAG, Er,Cr:YSGG, CO_2_, and Diode 980 nm)

**DOI:** 10.3390/ijerph17228665

**Published:** 2020-11-22

**Authors:** Samir Nammour, Marwan El Mobadder, Melanie Namour, Amaury Namour, Josep Arnabat-Dominguez, Kinga Grzech-Leśniak, Alain Vanheusden, Paolo Vescovi

**Affiliations:** 1 Department of Dental Science, Faculty of Medicine, University of Liege, 4000 Liege, Belgium; marwan.mobader@gmail.com (M.E.M.); melanienamour@gmail.com (M.N.); amaurynamour@gmail.com (A.N.); alain.vanheusden@chu.ulg.ac.be (A.V.); 2 School of Dentistry, University of Barcelona, 08907 Barcelona, Spain; 16324jad@comb.cat; 3 Dental Surgery Department, Medical University of Wroclaw, 50-425 Wroclaw, Poland; kgl@periocare.pl; 4 Department of Medicine and Surgery, University of Parma, 43121 Parma, Italy; paolo.vescovi@unipr.it

**Keywords:** capillary hemangioma, lasers, lip, oral pathology, oral surgery, venous lake, venous malformation

## Abstract

Different approaches with different clinical outcomes have been found in treating capillary hemangioma (CH), venous lake (VL), or venous malformations (VM) of the lips. This retrospective study aims to assess scar quality, recurrence rate, and patient satisfaction after different surgeries with different laser wavelengths. A total of 143 patients with CH or VM were included. Nd:YAG laser was used for 47 patients, diode 980 nm laser was used for 32 patients (treatments by transmucosal photo-thermo-coagulation), Er,Cr:YSSG laser was used for 12 patients (treatments by excision), and CO_2_ laser was used for 52 patients (treatments by photo-vaporization). The Manchester scar scale was used by practitioners to assess the scar quality. The recurrence rate and patients’ satisfaction were noted at different follow-ups during 12 months. Our retrospective study showed that laser-assisted aesthetic treatment of vascular lesions (CH, VL, and VM) of the lips can be considered effective regardless of the wavelength used (Er,Cr:YSGG, CO_2_, Nd:YAG, and diode 980 nm) or the treatment procedure (transmucosal photo-thermo-coagulation, photo-vaporization, and surgical excision). There was no significant difference in patient and practitioner satisfaction with aesthetic outcome at 6 months follow-up. Furthermore, the treatments of lip vascular lesions performed using Er,Cr:YSGG and CO_2_ lasers did not show any recurrence during the 12 months of follow-up, while recurrence rates of 11% ± 1.4% and 8% ± 0.9% were seen in the diode and Nd:YAG groups, respectively.

## 1. Introduction

Vascular lesions of the head and neck present a broad pathological spectrum, with a diversity of tumors and malformations including simple capillary irregularities and complex irregularities of the arteries, veins, and lymphatics [1]. In the literature, numerous classifications have been found to describe the vascular lesions; one of the most admitted classifications is that of the International Society for the Study of Vascular Anomalies (ISSVA) updated in 2014 [2]. According to this classification, these lesions are grouped as either vascular tumors, characterized by a proliferation of blood vessels, or vascular malformations, characterized by vessels with abnormal structure [2].

Capillary hemangioma (CH) is a benign vascular tumor that appears clinically as a raised, red area found anywhere in the body with a prevalence of 83% in the head and neck region [2,3]. On the other hand, venous malformations (VM) represent another frequent vascular lesion of the head and neck region with a prevalence of 50% and with a clinical aspect of soft, compressible, non-pulsatile blue-violaceous papules or nodules that can increase in size [2,3]. Furthermore, venous lake (VL) is a common benign vascular lesion defined as vascular ectasias formed from dilated venules located in the upper dermis [2,3,4].

Patients with capillary hemangioma and venous lip malformation usually seek treatment for at least one of the following reasons: aesthetic alteration, functional limitations, and psychological concerns. These vascular lesions can sometimes be subjected to a tendency of spontaneous regression; thus, the treatment approach depends largely on several factors that characterize each case. These factors are essentially size, location, and behavior of the lesion, as well as age and systematic condition of the patient [5,6].

The total excision of localized, small, and discrete capillary hemangioma and venous malformation is considered as the treatment of choice [5,6]. During surgical removal of these lesions, special care and precautions must be taken into consideration in order to avoid recurrence, as it has been found that these lesions have a tendency of recanalization. On the other hand, care must likewise be taken in order to avoid any irreversible unaesthetic outcomes, especially when these vascular lesions are localized in aesthetically sensible areas [6,7].

One of the first indications of laser in dermatology and dentistry is the management of vascular lesions [8]. Wavelengths with selective tissue absorption and deeper penetrations such as near-infrared lasers, specifically, Nd:YAG and diode lasers, are generally indicated for CH and VM [8,9]. In addition, the application of photo-selective absorption wavelengths [10,11,12,13] such as alexandrite lasers and other devices were reported to have a promising aesthetic outcome [14].

Despite the promising approach of these lasers toward the vascular lesions, there is not enough literature concerning the best-indicated wavelength commonly used in dentistry, the long-term aesthetic results, and the optimal treatment modality (excision, transmucosal photo-thermo-coagulation, and photo-vaporization).

The aim of this retrospective study was to evaluate the aesthetic outcome and the recurrence rates of capillary hemangioma, venous lake, and venous malformations of the lip performed using different surgical procedures and different laser wavelengths (Nd:YAG, Er,Cr:YSGG, CO_2_, and diode 980 nm lasers). The null hypothesis was that there is a difference in the long-term aesthetic outcome across different protocols.

## 2. Material and Methods

### 2.1. Study Design

This multicenter retrospective study was carried out using data collected in the period between 2004 and 2019. The data collection was made for all capillary hemangiomas (CHs) of the lip, as well as all venous lake and venous malformations (VMs) of the lip, treated with one of the following laser wavelengths: neodymium-doped yttrium, aluminum, and garnet laser (Nd:YAG; 1064 nm), erbium/chromium-doped yttrium, scandium, gallium, and garnet (Er,Cr:YSGG laser; 2790 nm), carbon dioxide laser (CO_2_; 10,600 nm), and diode laser (980 nm). Furthermore, according to the ethical committee recommendations of our university hospitals, the decision for surgery was made after informing all participating patients about the steps of the surgery, the risks, and the possible postoperative discomfort and complications. The surgery was performed after receiving a written informed consent form signed by the patient. Concerning data collection for retrospective analysis this was not considered as a new clinical study and did not require any approval from the ethical committee of the University of Liege.

### 2.2. Participants

A total of 143 patients participated in this retrospective study; the mean age of the patients was 48 (43–74), with 56% females (*n* = 81) and 44% males (*n* = 62). The average size of the lesions was 6 mm with a maximum of 15 and a minimum of three (Table 1). Specifically, 46 patients were treated with the Nd:YAG laser (*n* = 47), 12 were treated with the Er,Cr:YSSG laser (*n* = 12), 52 were treated with the CO_2_ laser (*n* = 52), and 32 were treated with the diode laser (*n* = 32) (Table 2). Diagnosis of the included capillary hemangioma and venous lake or venous malformation of the lip was based on a meticulous clinical exam and detailed medical history (date of appearance, growth, associated symptoms). In addition, in 29 cases, color Doppler ultrasonography was used to obtain additional information related to the vascularization, the flow type, the location, the three-dimensional (3D) profile, the type of vascular pedicles, and the lesion volume. A graded periodontal probe was used to evaluate the size of the lesions. The data were retrospectively entered into the database including patient demographics (age, gender, anatomical region, dimension of the lesion), type of lesion (i.e., capillary hemangioma or venous lip malformation), type of laser used (Nd:YAG, Er,Cr: YSGG, CO_2_, or diode), and the Manchester scar score (MSS) used in the postoperative follow-up (Table 3) [15]. Additionally, the visual analogue scale (VAS) was used in order to assess patient satisfaction. The VAS consisted of numbers from 0–10, where 0 represented “the worst satisfaction possible” and 10 represented “the greatest satisfaction possible”. All data were eventually collected. The follow-up periods for additional assessments of MSS and VAS were carried out at 2 weeks, 1 month, 6 months, and 12 months after the treatments.

#### Inclusion and Exclusion Criteria

Patients complaining of an unaesthetic capillary hemangioma, venous lake, and/or venous lip malformation were included. The exclusion criteria were chronic diseases, diabetes, immunosuppressed patients, and the existence of other benign or malign tumors in the same area.

### 2.3. Surgical Techniques

Each treatment was made with one of the following lasers: Nd:YAG, Er,Cr:YSGG, CO_2_, and diode. Local infiltration of anesthesia covering the area of the lesion was made before any surgical treatment. Prior to surgery, the operator, assistants, and patient wore specific eyeglasses for each wavelength, and all safety measures for the use of lasers were taken. After the treatment, all wounds were left to heal without any sutures. The collected data were divided into treatments performed by transmucosal photo-thermo-coagulation, photo-vaporization, and surgical excision. Specifically, nonsurgical procedures of photocoagulation were performed using the diode and Nd:YAG lasers, while photo-vaporization of the lesions was carried out using the CO_2_ laser, and excision of the lesions was performed using the Er:YAG laser because of its explosive mechanism and its difficulty to vaporize blood without destroying the mucosal layer covering blood cisterns.

#### 2.3.1. Er,Cr:YSGG Laser

**Figure 1:**
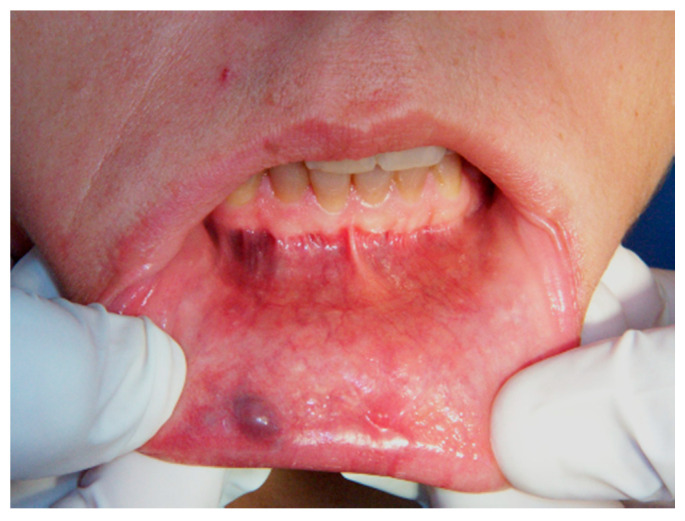
Clinical aspect of venous malformation (VM) on the lower lip.

**Figure 2:**
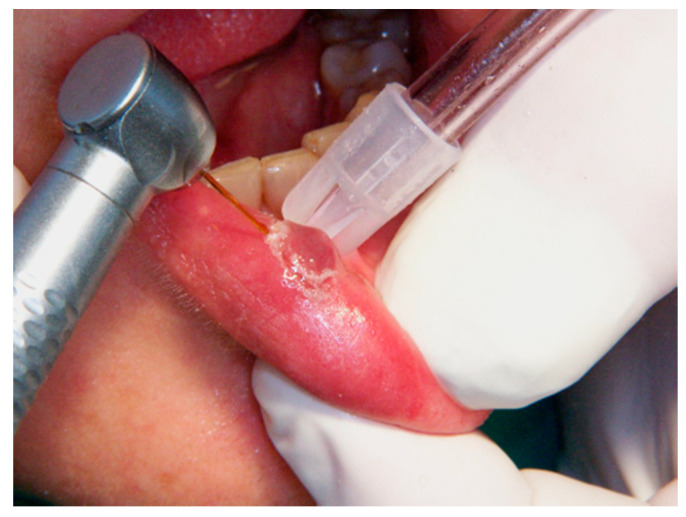
The lesion’s resection limit is indicated by the Er,Cr:YSGG laser around the surgical site.

**Figure 3:**
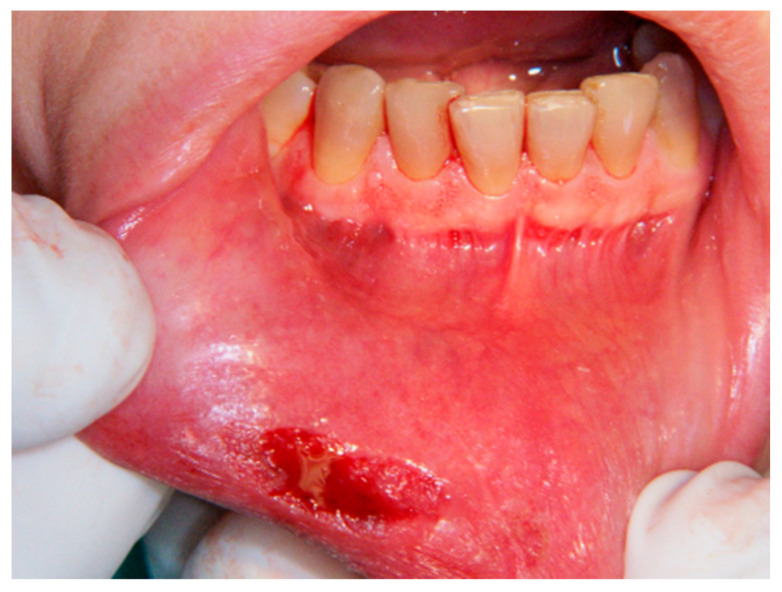
Bleeding which appeared after lesion excision.

An Er,Cr:YSGG laser with a wavelength of 2790 nm (Waterlase, Biolase Inc., Foothill Ranch, CA, USA) was used. After the injection of local anesthesia, the excision areal limit was delimited by the laser beam without air–water spray with an output power of 0.25 W and 20 Hz (Figure 1, Figure 2 and Figure 3). An excision was made for all lesions with an output power of 1.5 to 2 W (27 J/cm^2^), a fiber diameter of 600 μm, and pulsed mode (20 Hz, 60 μs). In addition, a defocused (noncontact) mode without air–water spray was used for the coagulation of the bottom of the wound at the end of the excision to ensure a primary coagulation. The wound was left to heal in the second intention, and no sutures were made.

#### 2.3.2. CO_2_ Laser

**Figure 4:**
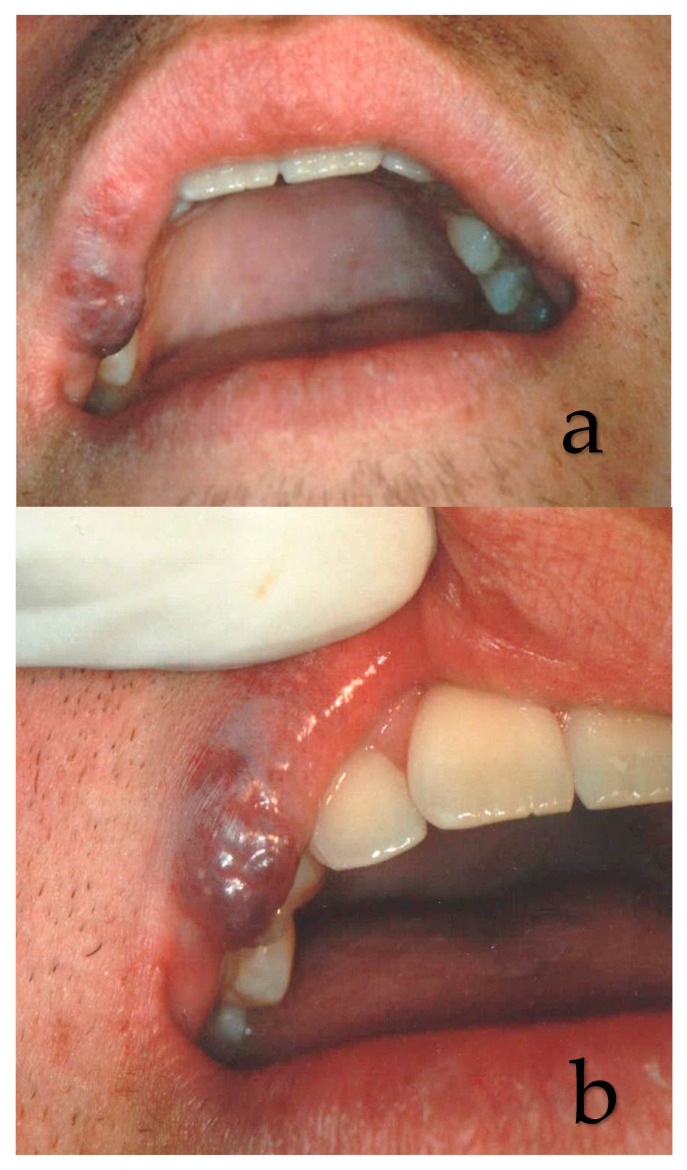
(**a**,**b**) Clinical aspects of capillary hemangioma (CH) of the upper lip. The lesion was a result of a trauma.

**Figure 5:**
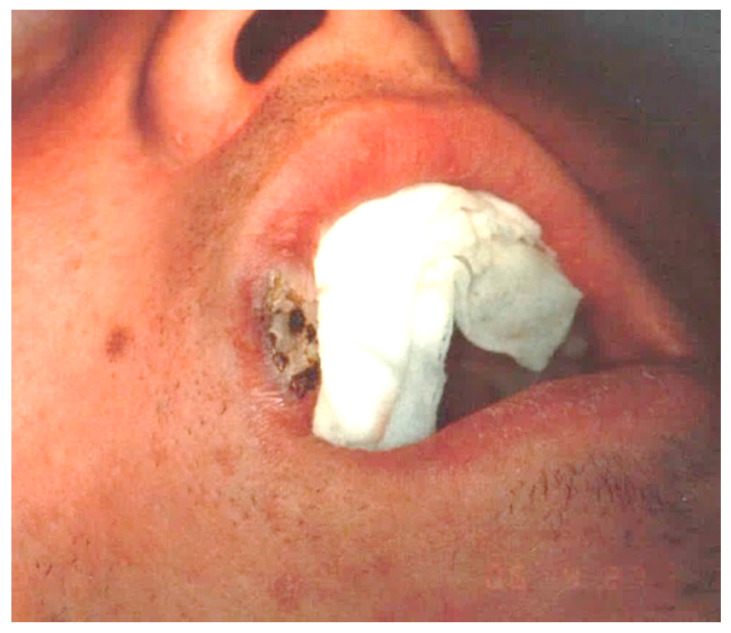
Vaporization of the lesion in defocused mode using the CO_2_ laser.

**Figure 6:**
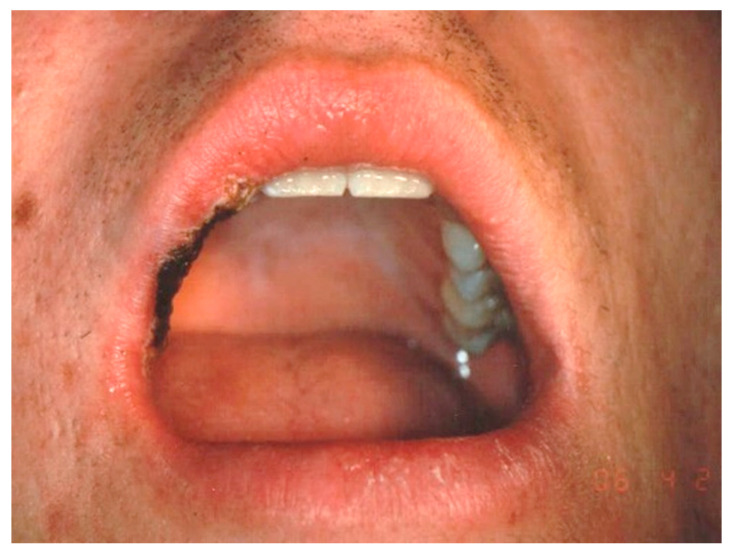
The mucosal layer covering the blood cittern was vaporized and removed in focused mode using the CO_2_ laser.

A CO_2_ laser with a wavelength of 10,600 nm (Smart US20 D Laser, High Tech Laser, Herzele, Belgium) was used. After injection of local anesthesia, the vascular lesion was photo-vaporized in a defocused (noncontact), continuous wave (CW), with an output power of 1 W (149.1 J/cm^2^ at focal point). The beam diameter was adapted to the diameter of each lesion minus 1 mm (−1 mm) in order to avoid irradiation of healthy tissue surrounding the lesion. The purpose was to generate heat inside tumors to vaporize the water inside the lesion, without allowing vaporization of the superficial layer of the mucosa covering the blood citterns (Figure 4a,b and Figure 5). After complete vaporization of the blood inside the lesion, the external layer of the mucosa was vaporized and removed in focused mode until complete exposure of the tumor cavity. The blood coagulation at the bottom of the citterns was performed in defocused mode (Figure 6). The surgical procedure was considered done after verification of hemostasis via simple pressure on the operated site. The wound was left to heal in the second intention, and no sutures were made.

#### 2.3.3. Nd:YAG Laser Irradiation

**Figure 7:**
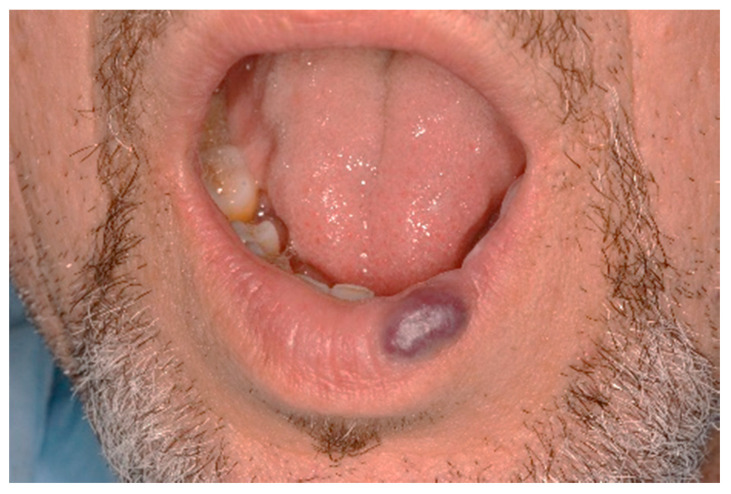
Clinical aspect of the lesion VM on the lower lip.

**Figure 8:**
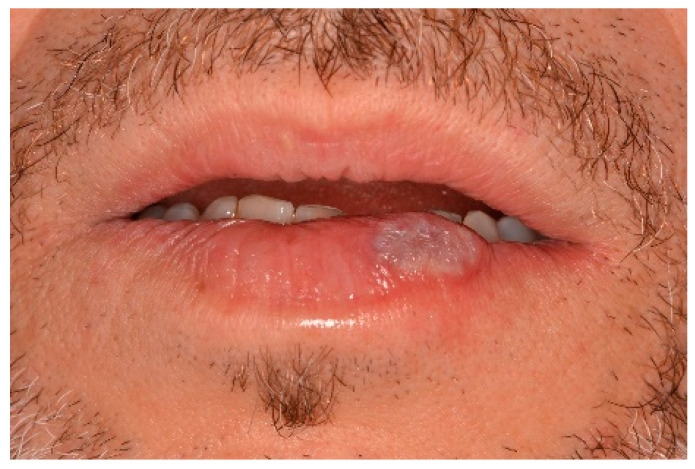
The lesion was treated by transmucosal photo-thermo-coagulation after topical anesthesia application. An Nd:YAG laser was used.

An Nd:YAG laser with a wavelength of 1064 nm (Fidelus plus, Fotona medical laser, Ljubljana, Slovenia) was used. After topical application of local anesthesia, a transmucosal photo-thermo-coagulation was made in noncontact mode, with an output power of 2 Watts, fiber diameter of 320 μm, pulsed mode (15 Hz), pulse duration of 320 µs (LP mode), and an energy density of 11.94 J/cm^2^. The lesion was treated by transmucosal photo-thermo-coagulation, and the treatment was considered completed when a blanching and visible shrinkage of the blood appeared inside the CV or VM (Figure 7 and Figure 8). When necessary, a second passage of the photo-vaporization cycle was performed after 2 weeks in cases of persistence or recurrence of the lesion. The wound was left to heal without any other treatment, and no sutures were made.

#### 2.3.4. Diode Laser

A diode laser with a wavelength of 980 nm (Smart M Pro, Lasotronix, Poland) was used. In order to avoid irradiation of healthy tissue surrounding the lesion, the diameter of the laser beam was changed depending on the size of the lesion so that the diameter of the beam was always about 1 mm less than the diameter of the lesion. After topical application of local anesthesia, the laser’s tip was not activated, and the irradiation was carried out in noncontact mode at an average distance of almost 2 mm in a continuous wave (CW). The irradiation conditions were an output power of 4 W (248.5 J/cm^2^) and fiber diameter of 320 µm. Similarly to the Nd:YAG procedure, transmucosal photo-thermo-coagulation was carried out after the topical application of local anesthesia. In cases of persistence or recurrence of a reduced size of the lesion, repetition of the treatment was performed after 1 or 2 weeks. The wound was left to heal without sutures.

#### 2.3.5. Postoperative Instructions and Recommendations

After each treatment, each patient was prescribed a painkiller and a topical oral disinfecting solution. In addition, instructions were given to patients to avoid eating hard or hot foods for the first 5 days after the procedure.

### 2.4. Evaluation of the Aesthetic Results

In order to evaluate the aesthetic outcome of the treatment and in order to transcribe any potential complications or compromise in the aesthetic outcome after wound healing, the Manchester scar scale (MSS) was used for practitioners’ evaluations (Table 1). On the other hand, in order to evaluate patient satisfaction, a visual analogue scale was used with values from 0–10 where 0 represented “the worst satisfaction possible” and 10 represented “the highest satisfaction possible”. The follow-up periods were assigned 2 weeks, 1 month, 6 months, and 12 months after the treatment.

### 2.5. Evaluation of the Recurrence Rate

A qualitative visual evaluation was made for the recurrence of CH and VM after the treatment. The patients were asked to immediately contact the proper practitioners if any aspect of capillary lesion or abnormality was seen on the treated lip. Any reappearance or recurrence of the lesion was noted.

### 2.6. Statistical Analysis

For statistical analysis, Prism 5^®^ software (GraphPad Software, Inc., San Diego, CA, USA) was used. The confidence level of the study was proposed to be 95% with a *p*-value < 0.05 considered as statistically significant for the analysis. Descriptive statistics, including the means and standard deviations, were also calculated. One-way ANOVA coupled with a Newman–Keuls multiple comparison test (post hoc test) was used.

## 3. Results

### 3.1. Results of the Manchester Scar Scale Evaluation

The global mean average of the Manchester scar scale after 12 months was significantly lower (*p* < 0.05) compared to the results obtained just after 2 weeks of treatment for all wavelengths. Furthermore, regardless of the laser wavelength and regardless of the surgical approach (transmucosal photo-thermo-coagulation, photo-vaporization, and surgical excision), the healing process (the quality of the scar) was acceptable and satisfactory after 6 months of follow-up in all groups (Table 4).

**Figure 9:**
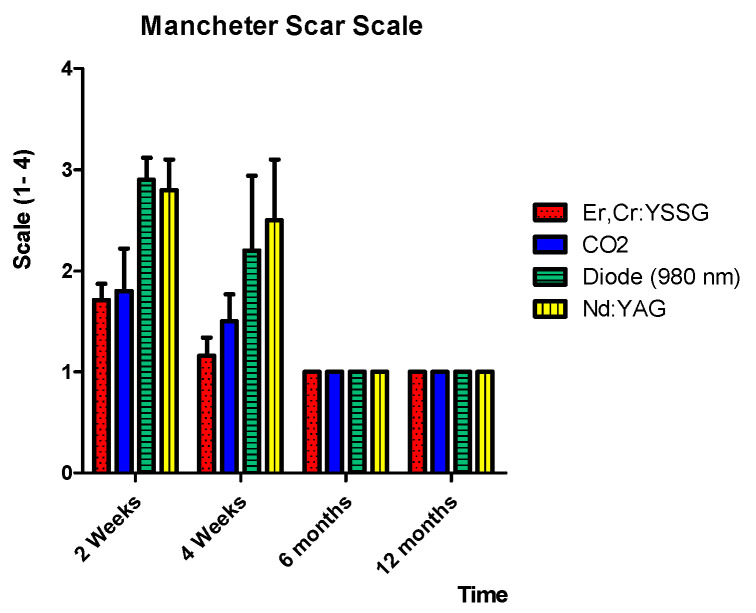
Global mean values of the Manchester scar scale for the different laser wavelengths and at different follow-ups.

After 2 weeks of follow-up, the Er,Cr:YSSG (1.71 ± 0.16) and the CO_2_ (1.8 ± 0.42) lasers resulted in significantly better scar quality compared to the Nd:YAG laser (2.8 ± 0.30) and diode laser (2.9 ± 0.22) (Figure 9). Only the Er,Cr:YSSG group showed no statistically significant difference at 1 month of follow-up (1.16 ± 0.18) and above (1).

### 3.2. Recurrence Rate

It was necessary to have a second treatment session in groups treated with the diode and Nd:YAG lasers. Recurrence with obligation to perform a second treatment session was observed in 11% ± 1.4% of cases treated in the diode laser group and 8% ± 0.9% of cases treated in the Nd:YAG laser group. On the other hand, in the Er,Cr:YSSG and CO_2_ groups, no recurrence was noted after the first treatment sessions (Table 5).

### 3.3. Patient Satisfaction

**Figure 10:**
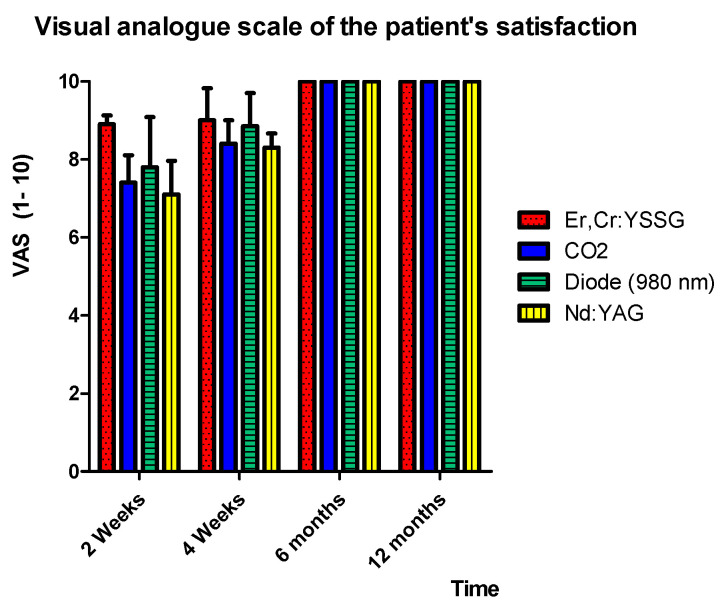
Visual analogue scale of patient satisfaction at different follow-ups. Zero represents the worst satisfaction possible and 10 represents the highest satisfaction possible.

After 2 weeks, the highest values of satisfaction was obtained with the Er,Cr:YSSG laser (8.9 ± 0.23), followed by the diode laser (7.8 ± 1.58), CO_2_ laser (7.4 ± 0.7), and Nd:YAG laser (7.10 ± 0.86) (Table 6). The difference between the values of each group was statistically significant (*p* < 0.05). The values significantly increased with follow-up for all groups, attaining the highest satisfactory score (±10) at 6 months. Therefore, it can be considered that, regardless of the wavelength, patients showed satisfaction with regard to the aesthetic outcome within 6 months of follow-up (±10) (Table 6, Figure 10).

**Figure 11:**
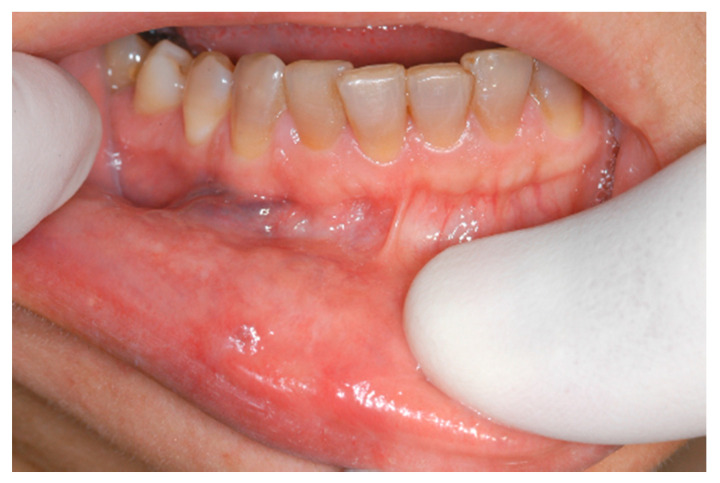
The aspect of healing at 2 weeks post operation. The excision was performed using an Er,Cr:YSSG laser.

**Figure 12:**
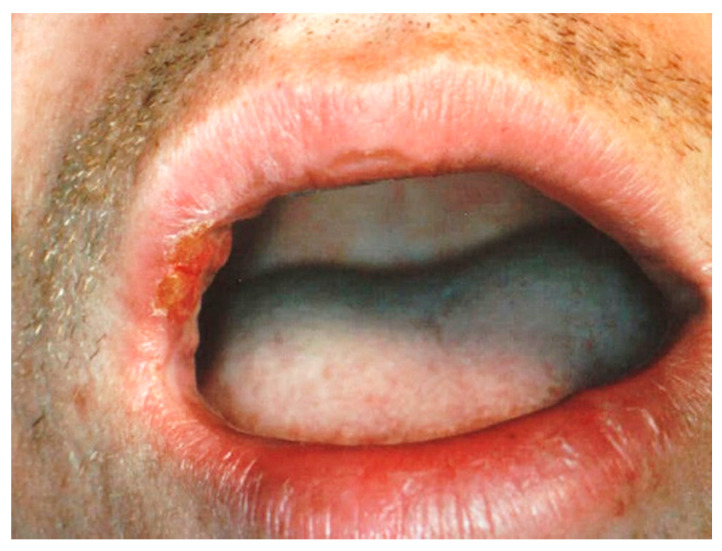
View of the healed area at 2 weeks post operation. The vaporization was performed using a CO_2_ laser.

**Figure 13:**
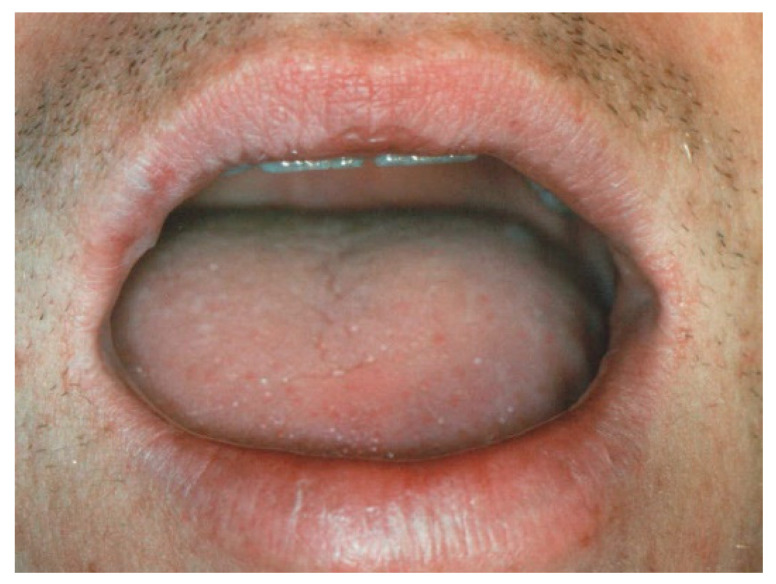
Clinical aspect of the healed area at 4 weeks post operation. The vaporization was performed using a CO_2_ laser.

**Figure 14:**
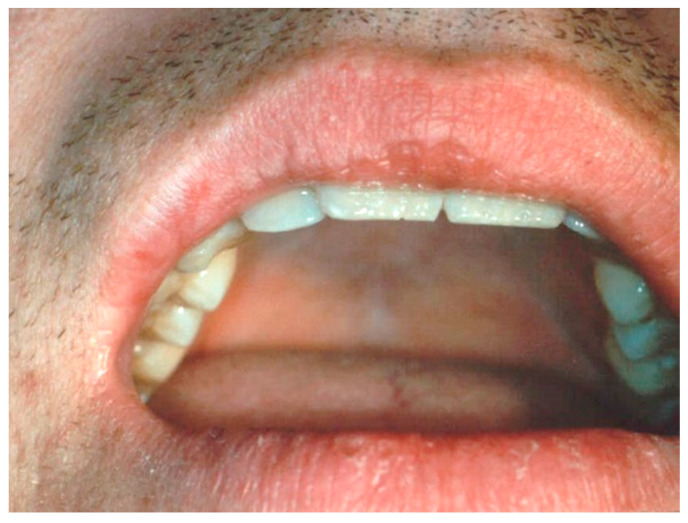
The aesthetic aspect at 6 months post operation. The vaporization was performed using a CO_2_ laser.

**Figure 15:**
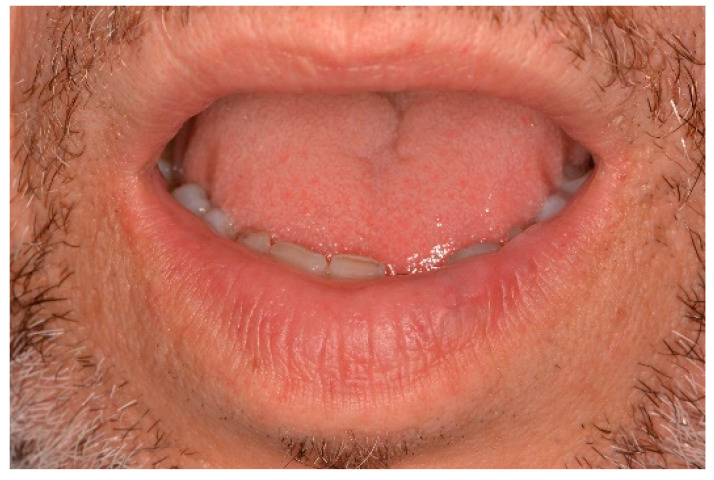
Clinical aspect of healing at 2 weeks post operation. Transmucosal photo-thermo-coagulation was performed using an Nd:>YAG laser.

The aesthetic appearance of the scarred treated areas varied depending on the surgery performed at each wavelength (Figure 11, Figure 12, Figure 13, Figure 14 and Figure 15).

## 4. Discussion

The use of laser beams for the management of vascular lesions dates back to the 1960s [16,17,18]. Back then, ruby and argon lasers were introduced to improve the color of hemangiomas and post-wine stains. However, their nonselective photothermolysis of the targeted tissue resulted in frequent negative postoperative scarring and pigmentation changes [19,20,21,22]. After the vast development of lasers and after improvements in the understanding of laser–tissue interactions and laser physics, the treatment of vascular lesions is considered as one of the most common indications of laser [23,24,25,26,27].

This multicentric retrospective study showed that the treatment outcome of capillary hemangioma, venous lake, and venous malformation of the lip with different wavelengths was reported to be successful, and both patients and operators reported satisfaction concerning the aesthetic outcome at 6 months and above of follow-up. In addition, it was revealed that the quality of the scar (the quality of the healing) after 4 weeks was higher in the group treated using the Er,Cr:YSSG and CO_2_ lasers showing an MSS of 1.16 ± 0.18 and 1.5 ± 0.27, respectively. The aesthetic outcome values obtained with the Er,Cr:YSSG (incision) and CO_2_ (total photo-vaporization of lesions) lasers were significantly better than those obtained with the diode and the Nd:YAG lasers (transmucosal photo-thermo-coagulation). Consequently, the Er,Cr:YSSG and CO_2_ lasers gave better scar quality at 2 weeks and 4 weeks after treatment. However, at 6 months and above, all surgical procedures and wavelengths showed similar satisfactory quality of the scar. Furthermore, the aesthetic quality of scars resulting from the diode and Nd:YAG lasers was low until 1 month of follow-up, but gave satisfactory results similar to other wavelengths after 6 months of follow-up.

In this retrospective study, the Manchester scar scale (MSS), proposed in 1998 by Beausang et al. was used because of its detailed and relevant description of the color, texture, size, and margin of the scar and its association with the surrounding tissue [15]. In addition to the MSS, a patient-based visual analogue scale was used to assess the patient’s perception of the healing aspect. Furthermore, it was noted that there is practically no standardized methodology and systematic approach for the assessment of scars and the quality of healing in post-operative surgical sites [28].

The nonsurgical procedure of transmucosal photo-thermo-coagulation applied using the diode and Nd:YAG lasers consists of targeting the chromophores inside the vascular lesions, essentially hemoglobin [29,30,31]. These chromophores absorb the laser’s energy and convert it into heat, which is transferred to the vessel wall, causing coagulation and vessel closure and, finally, thrombosis of the blood vessels [32]. Concerning photo-vaporization using the CO_2_ laser, the absorption of photons by the water content of the tumor provokes a sudden local increase in temperature resulting from vaporization of the blood cittern. At the end of the surgery, the mucosal layer covering the blood cittern is totally vaporized, exposing the bottom of the tumor cavity [33,34,35]. On the other hand, the Er,Cr:YSSG laser procedure consists of complete excision of the vascular lesion, followed by coagulation of the bottom of the wound for primary hemostasis [36,37].

A large number of approaches are described in literature for the management of vascular lesions [8,38,39,40]. These methods principally depend on the type, severity, size, location, and possible complications of the vascular lesion [14,38,39,40,41,42,43].

John et al. conducted a study on the treatment of venous lesions of the lips with a long-pulsed Nd:YAG laser. The study included 31 patients with venous lesions of the lip [44]. The parameters used were spot size depending on the size of the lesion, variable energy density with an average of 80 J/cm^2^, and pulse width of 20. They concluded that the treatment was effective with 87% of patients having no recurrence and one patient having a small, contracted scar [44]. In contrast to John et al., the recurrence rate in our study was lower (only ±8%) with the Nd:YAG laser and no contracted scar was seen. Scherer et al. also studied the use of the Nd:YAG laser for venous malformations of the face, including 146 patients [45]. In their study, the Nd:YAG laser was used alone if the lesion was considered relatively small or followed by a surgical excision if the lesion was considered deep. In addition, a retrospective study by Mungnirandr et al. assessed the safety and effectiveness of the Nd:YAG laser for venous malformation in the oral cavity [46]. The study included 10 children with inoperable VM and established that the Nd:YAG laser is a promising alternative treatment in pediatric patients with venous malformations in the oral cavity, whereby oral complications mostly seen involved mild to moderate scarring [46]. Interestingly, the treatment protocol used in their study included the use of the Nd:YAG in contact mode, in noncontact mode, and using interstitial techniques. The choice of each technique depended essentially on the size and depth of the lesions. Specifically, the contact technique involved placement of the fiber-optic end of the laser handpiece directly against the mucosal surface during laser irradiation. The noncontact process, however, involved the laser beam being emitted through a clear ice cube for purposes of minimizing epidermal injury [47]. The interstitial technique required the insertion of the fiber-optic end of the handpiece during laser irradiation inside the lesion [46].

It can be found in the literature that most treatments for vascular lesions were performed with the Nd:YAG laser, albeit using different wavelengths. Del Pozo et al. conducted a study with a CO_2_ laser on five cases of venous malformations with lip involvement [47]. The study revealed that vaporization with the carbon dioxide laser was able to flatten the surface of the lip and, therefore, was considered an effective palliative treatment of lip venous malformations [47]. Compared to our study, the treatment protocol with the CO_2_ laser was different. Del Pozo et al. irradiated the treatment area with several passages in contact mode to achieve a contraction and immediate flattering of the lesion surface. However, in our study, noncontact mode was used in order to generate heat to vaporize the water inside the vascular lesion; then, the external layer of the mucosa was vaporized and removed in focused mode until complete exposure of the lesion bottom. Unlike our study, there was no recurrence noted in Del Pozo’s study; however, their purpose was not to completely remove the venous malformation but to shrink its size. Bacci et al. performed a retrospective study on the use of a diode laser to treat small oral vascular malformations. The study included 59 patients, which showed that the only complication related to the surgery was modest pain. This pain 30 days after the reduction of the lesions, however, was considered as excellent or good in 52 cases and fair or poor in seven cases, whereas six patients required a second diode laser application [48]. The study revealed that the diode laser is considered acceptable for the treatment of small oral venous malformations and venous lakes with shorter postoperative complications and shorter operating times when compared to the conventional scalpel surgery [48]. Furthermore, Angiero et al. conducted a study on head and neck vascular lesions in pediatric patients treated with an endolesional 980 nm diode laser [48]. The study included 160 patients with hemangiomas, 50 with vascular malformations, and 40 with lymphatic malformations. The treatment results were analyzed by evaluating the decrease in lesion size and its complete clinical disappearance. All patients had a complete resolution of the vascular lesion except for 38, for which an additional session was required [48].

Topical therapy such as timolol, high-potency topical corticosteroids, imiquimod, and becaplermin gel are also recommended for superficial hemangiomas [40,41,42]. In fact, timolol has emerged as the preferred topical treatment. On the other hand, Lee et al. concluded, in a study on 11 patients with lip hemangioma, that the use of Dermabond after surgical excision prevents wound contamination and yields acceptable aesthetic results [43].

Since different operators used the Manchester scar scale (MSS) to assess the aesthetic outcome, a possible limitation of our study was the slight subjectivity obtained when different operators used the MSS.

Our retrospective study compared four laser wavelengths in the management of venous malformation and capillary hemangioma of the lip with three different surgical approaches (excision, photo-vaporization, and transmucosal photo-thermo-coagulation). Further studies are necessary to compare the aesthetic outcome, the recurrence rates, and the possible postoperative complications of different protocols.

The null hypothesis was rejected because no statistical difference in the aesthetic outcome at the end of follow-up was obtained by the three different surgical procedures, i.e., transmucosal photo-thermo-coagulation (Nd:YAG and diode lasers), photo-vaporization (CO_2_ laser), and surgical excision (Er,Cr:YSGG laser).

## 5. Conclusions

Our retrospective study showed that laser-assisted aesthetic treatment of vascular lesions of the lips can be considered effective regardless of the wavelength used (Er,Cr:YSGG, CO_2_, Nd:YAG, and diode lasers) or the treatment procedure (transmucosal photo-thermo-coagulation, photo-vaporization, and surgical excision). There was no significant difference in patient and practitioner satisfaction with aesthetic outcome at 6 months follow-up. Furthermore, the treatments of lip vascular lesions performed using Er,Cr:YSGG and CO_2_ lasers did not show any recurrence during the 12 months of follow-up, while recurrence rates of 11% ± 1.4% and 8% ± 0.9% were seen in the diode and Nd:YAG groups, respectively.

## Figures and Tables

**Table 1:** Clinical features of the treated patients.

Total	Gender		Age Range (years)	Lesion Diameter (mm)		
	Female	Male		Average	Minimum	Maximum
143	56%	44%	48	6	3	15
	(*n* = 81)	(*n* = 62)	(min: 43; max: 74)			

Age in years; lesion diameter in millimeters; min = minimum; max = maximum.

**Table 2:** Repartition of the type of laser used for each patient.

Laser Wavelength (nm)	Number of Patients
Nd:YAG, 1064 nm	47
Er,Cr:YSSG, 2780 nm	12
CO_2_, 10,600 nm	52
Diode laser, 980 nm	32
	143

**Table 3:** Description of the Manchester scar scale [15].

Description	Score
*Color*	
Perfect	1
Slight mismatch	2
Obvious mismatch	3
Gross mismatch	4
*Matte* vs. *Shiny*	
Matte	1
Shiny	2
*Contour*	
Flush with surrounding skin	1
Slightly proud/indented	2
Hypertrophic	3
Keloid	4
*Distortion*	
None	1
Mild	2
Moderate	3
Severe	4
*Texture*	
Normal	1
Just palpable	2
Firm	3
Hard	4

**Table 4:** Global mean value of the Manchester scar score at different follow-ups as a function of the laser type.

Laser Wavelength	MSS at Different Time of Follow-Up			
	2 Weeks	1 Month	6 Months	12 Months
Er,Cr:YSSG	1.71 ± 0.16 ^A^	1. 16 ± 0.18 ^B^	1 ^B^	1 ^B^
CO_2_	1.8 ± 0.42 ^A^	1.5 ± 0.27 ^C^	1 ^B^	1 ^B^
Diode	2.9 ± 0.22 ^D^	2.2 ± 0.74 ^E^	1 ^B^	1 ^B^
Nd:YAG	2.8 ± 0.30 ^D^	2.5 ± 0.62 ^E^	1 ^B^	1 ^B^

The scale is based on a score from 1–4 where 1 represents an excellent result and 4 represents a poor. Identical superscript letters indicate no statistically significant difference, and different superscript letters indicate a statistically significant difference (*p* < 0.05). Refer to Table 1 for a descriptive explanation of the Manchester Scar Score.

**Table 5:** Recurrence rate of different surgical procedures.

Laser Wavelength	Er,Cr:YSSG (Excision)	CO_2_ (Photo-Vaporization)	Diode (Transmucosal Photo-Thermo-Coagulation)	Nd:YAG (Transmucosal Photo-Thermo-Coagulation)
Recurrence rate (%)	0 ^A^	0 ^A^	11% ±1.4% ^B^	8% ± 0.9% ^C^

Identical superscript letters indicate no statistically significant difference and different superscript letters indicate a statistically significant difference (*p* < 0.05).

**Table 6:** Visual analogue scale of patient satisfaction at different follow-ups.

Laser Wavelength	Visual Analogue at Different Time of Follow-Up			
	2 Weeks	1 Month	6 Months	12 Months
Er,Cr:YSSG	8.9 ± 0.23 ^A^	9 ± 0.82 ^A^	10 ^B^	10 ^B^
CO_2_	7.4 ± 0.7 ^C^	8.4 ± 0.6 ^D^	10 ^B^	10 ^B^
Diode	7.8 ± 1.58 ^E^	8.85 ± 0.85 ^A^	10 ^B^	10 ^B^
Nd:YAG	7.10 ± 0.86 ^F^	8.30 ± 0.36 ^D^	10 ^B^	10 ^B^

Zero represents the worst satisfaction possible, and 10 represents the highest satisfaction possible. Identical superscript letters indicate no statistically significant difference, and different superscript letters indicate a statistically significant difference (*p* < 0.05).
